# Supplementary figures and images for: Examination of Wnt signaling as a therapeutic target for pancreatic ductal adenocarcinoma (PDAC) using a pancreatic tumor organoid library (PTOL)
Source: PLoS One. 2024 Apr 10;19(4):e0298808. doi: 10.1371/journal.pone.0298808 (PMC11006186; doi:10.1371/journal.pone.0298808)

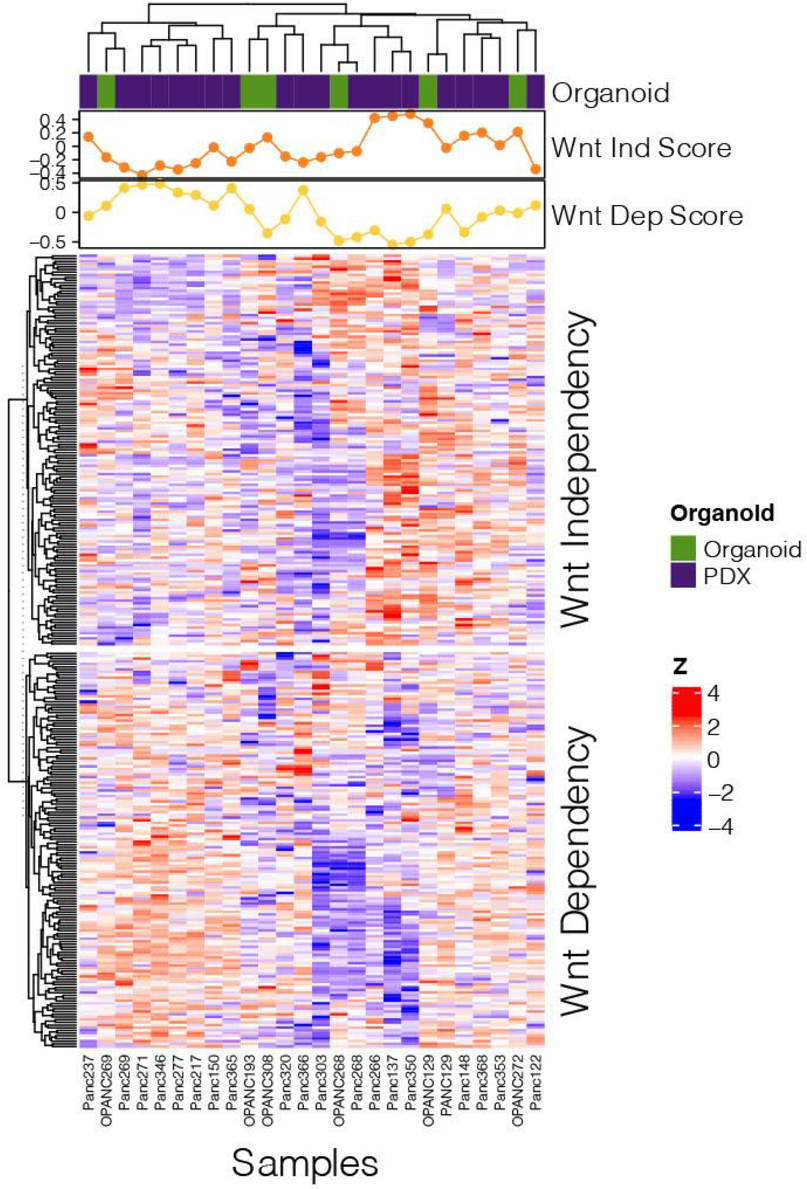

Supplement: S1 Fig — [1]. Heatmap displaying Wnt (in)dependent gene signatures for all PDX PDAC tumor and organoid lines. Gene names as describe by Seino et al referenced in S2 Table. The dependency scores are generated using GSVA. The CPM of each gene was z-score transformed across each row. Pancreatic organoid lines compared to their respective PDX tumors. OPANC = organoid, PANC = PDX tumor. (TIF) [file pone.0298808.s001.tif]

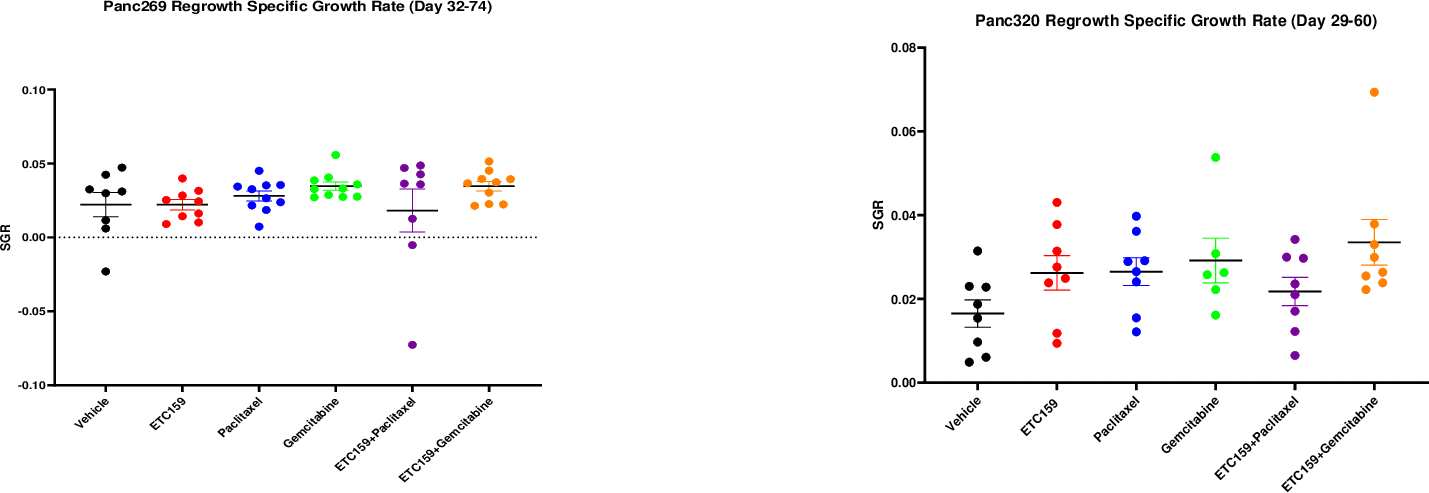

Supplement: S2 Fig — Athymic nude mice with subcutaneously injected tumors were treated for 30 days with single agent or combination of ETC-159 and either Paclitaxel or Gemcitabine. Specific growth rate was calculated, demonstrating more affective reduction of growth rate with combinatory treatment of Wnt dependent Panc269, consistent with in vitro data. Panc320 also demonstrated significant reduction in growth rate with combination of ETC-159 and Paclitaxel, but otherwise growth was not as affected in this Wnt independent model supported by in vitro data. (TIF) [file pone.0298808.s002.tif]

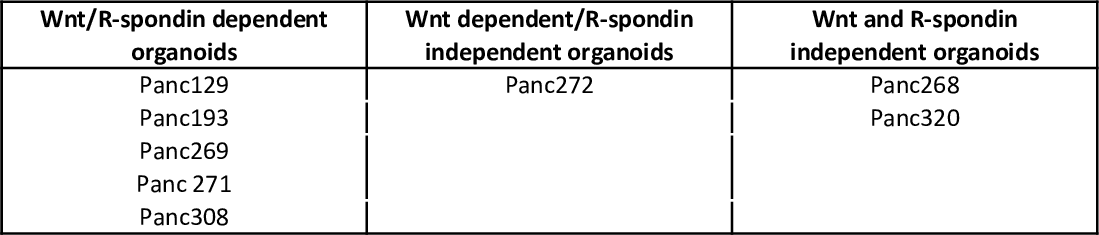

Supplement: S1 Table — (TIF) [file pone.0298808.s003.tif]
