# Supplementary material for: Examination of Wnt signaling as a therapeutic target for pancreatic ductal adenocarcinoma (PDAC) using a pancreatic tumor organoid library (PTOL)
Source: PLoS One. 2024 Apr 10;19(4):e0298808. doi: 10.1371/journal.pone.0298808 (PMC11006186; doi:10.1371/journal.pone.0298808)
Supplement: Attachment — Submitted filename: Review Comments.docx [file pone.0298808.s005.docx]

The manuscript ‘Examination of Wnt signaling as a therapeutic target for pancreatic ductal adenocarcinoma (PDAC) using a pancreatic tumor organoid library (PTOL)’ by Hawkins et al, studied the Wnt dependent and independent phenotypes of pancreatic tumors using organoids. This reviewer provides the following comments/suggestions to be addressed by the Authors.

1. Did the authors observed any correlation between the mutations identified in the organoids and niche factor dependency? Please explain in detail regarding the same in the manuscript.
2. No much difference in viability was observed between 1μM or 10 μM concentration of ETC-159 and C59. And for combination analysis, ETC-159 was used at concentrations 0.313 μM, 0.625 μM, 1.25 μM, 2.5 μM, 5 μM. Since there is no much effect on viability beyond 1 μM concentration, authors should consider using lower concentrations and a maximum of 1 μM. The concept of analyzing combinatorial drug concentrations is to check if we could decrease the drug concentration and increase the efficacy of treatment by using two different mode of actions. Authors have not mentioned at what combination dose the tumor growth rate was determined or for the gene expression analysis. Figure 4D is missing. On what basis authors have chosen paclitaxel and gemcitabine for combination with ETC-159? May be these two drugs do not have synergistic effect with ETC-159 or the doses used for combination are not appropriate.
3. If combination of paclitaxel and gemcitabine is already known and reported, then authors should have focused on identifying if ETC-159 and combination of paclitaxel and gemcitabine can have increased efficacy. Authors can make an attempt of this combination.
4. Colony formation can be represented as images
5. Authors should carefully go through the figures and tables provided, the supplement figure 2 contains supplement table 2.
6. Statistical analysis for all the graphs needs to be provided
