# Supplementary material for: Examination of Wnt signaling as a therapeutic target for pancreatic ductal adenocarcinoma (PDAC) using a pancreatic tumor organoid library (PTOL)
Source: PLoS One. 2024 Apr 10;19(4):e0298808. doi: 10.1371/journal.pone.0298808 (PMC11006186; doi:10.1371/journal.pone.0298808)
Supplement: Attachment — Submitted filename: Hayley J Hawkins et al.docx [file pone.0298808.s006.docx]

Hayley J Hawkins et al

Here are some constructive comments and observations you might consider for the provided based on the provided information about the research involving **PDAC (Pancreatic Ductal Adenocarcinoma) patient-derived samples, xenograft models, and organoid cultures,** here are some questions you might consider:

I. The text provides a detailed and structured overview of the methods and procedures employed in the study.
II. The division into sections like "Human Specimens," "PDAC Patient-Derived Xenografts," and "Pancreatic Tumor Organoid Library (PTOL) Generation and Characterization" helps organize the information logically.
III. The process of generating PDAC organoid lines is clearly outlined, including details on tissue preparation, digestion, culture conditions, and media components.
IV. Assessment of Wnt Dependency:
    a. the methodology for assessing the Wnt (in)dependency of PDAC tumor organoids is thoroughly explained, involving the manipulation of HPSC media and testing the effects on viability.
V. Wnt Inhibitor Studies:
   a. The steps involving the use of Porcupine and Wnt inhibitors to confirm Wnt (in)dependency are well-documented, including the criteria used to define dependency.
VI. **Minor Comments:**
    a. In certain sections, consider breaking down longer sentences for enhanced readability.
    b. For instance, consider using bullet points or subheadings for better organization in sections with multiple steps.
    c. The section on "Statistical Analysis of In vitro organoid drug treatment assays" is comprehensive but complex. Consider breaking down the information into subsections for clarity, separating details about individual drugs and combinations.
    d. Clarify the notation "SMAD4(2, 3)." If these numbers represent specific mutations or isoforms, provide a brief explanation. **Page 12 results section.**
    e. For better clarity, consider breaking the long sentence into two sentences: "Viability of Panc320 still required the presence of Noggin and A83-01. In contrast, Panc272 grew independently of all exogenous niche factors." **Page 12**
    f. Clarify the significance or implications of the observed changes in gene expression in Panc320 and Panc269.
